# The influence of early aging on eye movements during motor simulation

**DOI:** 10.1007/s11357-014-9671-y

**Published:** 2014-07-09

**Authors:** Sheree A. McCormick, Joe Causer, Paul S. Holmes

**Affiliations:** 1 Cognitive Motor Function Group, Institute for Performance Research, Manchester Metropolitan University Cheshire Faculty, Crewe Green Road, Crewe Cheshire, CW1 5DU UK; 2 Brain and Behaviour Laboratory, Liverpool John Moores University, Liverpool, UK

**Keywords:** Early aging, Eye movements, Motor simulation

## Abstract

Movement based interventions such as imagery and action observation are used increasingly to support physical rehabilitation of adults during early aging. The efficacy of these more covert approaches is based on an intuitively appealing assumption that movement execution, imagery and observation share neural substrate; alteration of one influences directly the function of the other two. Using eye movement metrics this paper reports findings that question the congruency of the three conditions. The data reveal that simulating movement through imagery and action observation may offer older adults movement practice conditions that are not constrained by the age-related decline observed in physical conditions. In addition, the findings provide support for action observation as a more effective technique for movement reproduction in comparison to imagery. This concern for imagery was also seen in the less congruent temporal relationship in movement time between imagery and movement execution suggesting imagery inaccuracy in early aging.

## Introduction

Covert training processes such as motor imagery (MI), the cognitive rehearsal of an action without actual execution (Denis 1985), and action observation (AO), the process of adapting action through observation learning (Bandura 1986), are increasingly proposed as adjuncts to physical therapy during the motor rehabilitation of older individuals (Ertelt et al. 2007; Page et al. 2007). The main tenet supporting the use of these motor simulation processes is that overt (action execution, AE) and covert (MI and AO) actions recruit similar, but not identical, cortical motor areas, and the activation of these motor areas, via any of the three processes, enhances brain plasticity (Jeannerod 1994; Rizzolatti et al. 1996). The use of MI and AO in clinical populations, however, has generally assumed that the motor simulation skills of older individuals are unaffected by age. In healthy aging, efficient movement can be compromised through: modifications within the musculoskeletal system (Smith et al. 1999; Kinoshita and Francis 1996); loss of sensorimotor and proprioceptive sensitivity (Klein et al. 2001; Leonard and Tremblay 2007; Skinner et al. 1984); a slowing in processing visual information (Briggs et al. 1999); or cognitive decline (Salthouse 1996). If, as neurophysiological studies increasingly demonstrate, the motor representation is shared between overt and covert conditions, then any detrimental age related changes associated with AE may also reduce the effectiveness and efficacy of the covert techniques.

The performance of overt and covert motor tasks is frequently compared using self-report inventories and brain mapping techniques. Although these are useful measures, self-reports rely on an individual’s introspective access to conscious awareness, and measures of neural activity do not provide data processing in real-time or instantaneous feedback (Collet et al. 2011). An alternative method, the chronometry paradigm, compares the time taken to perform and imagine a motor act, with similar movement time (MT) taken as evidence of imagery ability (Guillot and Collet 2005). An important aspect of this temporal relationship between AE and MI is that if task complexity is increased then MT increases in AE and MI (Decety et al. 1989). Thus both the physical and mental performance of action are governed by the speed-accuracy relationship known as Fitts’ Law (Fitts 1954). Researchers frequently exploit this phenomenon and use MT as a manipulation check to ensure task compliance in the covert tasks (Gabbard et al. 2011; Heremans et al. 2001; McCormick et al. 2012). The temporal correspondence between AE and MI is not suggested to develop until late adolescence, with proficiency achieved once the neural systems supporting internal modelling have matured (Caeyenberghs et al. 2009). Whilst older adults are reported to demonstrate temporal congruency (Sirigu et al. 1996), they may underestimate (Personnier et al. 2010; Skoura et al. 2005) and overestimate (Maruff et al. 1999; Skoura et al. 2008) the imagined MT. It is possible that the temporal inconsistency may be related to the task as younger individuals have been reported to over-estimate the imagined duration when more complex tasks are performed (Guillot and Collet 2005). The temporal inconsistency may, however, also reflect age-related changes in the cognitive mechanisms mediating the relationship between physical and mental practice. Objectively investigating online cognitive processing during these tasks may offer a more comprehensive method of comparing overt and covert performance in this age group. One method of achieving this is by measuring eye movements (Heremans et al. 2008; McCormick et al. 2013).

The gaze control system comprises mechanisms concerned with the acquisition of visually presented information, making it an excellent reflector of cognitive processes, including decision-making and attention (Sirevaag and Stern 2000). Although the extent to which gaze behavior represents the amount of cognitive processing has been questioned (e.g. Posner and Raichle 1994; Viviani 1990), recent research suggests that it is difficult to shift the point of gaze without shifting attention (Shinoda et al. 2001). The attention shifts that precede saccadic eye movements are associated with their preparation and involve some of the same neuronal “machinery” (Corbetta et al. 1998; Culham et al. 1998). Corbetta et al. (1998) examined fMRI and surface-based representations of brain activity to compare the functional anatomy of two tasks, one involving covert shifts of attention to peripheral visual stimuli, the other involving both attentional and saccadic shifts to the same stimuli. Overlapping regional networks in parietal, frontal, and temporal lobes were active in both tasks. This anatomical overlap is consistent with the hypothesis that attentional and oculomotor processes are tightly integrated at the neural level. Motter and Belky (1998) and Findlay and Gilchrist (1998) have also argued that fixations reflect attentional distribution in visual search experiments.

Contemporary research has compared the cognitive organization of an action in AE, AO and MI through the measurement of visual fixations (for a review see: Causer et al. 2013). These specific gaze parameters have been extensively used by cognitive and sport psychologists to infer the focus of attention (Causer et al. 2010; Hayhoe 2004). The number and spatial distribution of fixations is considered to reflect the visual information that an individual considers most important, the temporal distribution may be used to identify the relationship between the visual cues, and the duration is considered a measure of information processing demand (Zelinsky 2013). Using a reach and point task with high spatiotemporal demand, McCormick et al. (2013) reported that healthy, young adults attend the same visual cues in AE, AO and MI but that the visual information processing demand was congruent between AE and AO only. These findings highlight the sensitivity of using this method and suggest that there are discrete differences, as well as similarities, in the cognitive organization of overt and covert action, even in the absence of age related influences.

At present there appears a lack of research that has used eye movements to compare the cognitive processes of healthy, older adults during AE, MI and AO. In studies that have examined eye movements in AE, older adults are reported to need more time to extract and process the visual information and programme the appropriate motor responses (Di Fabio et al. 2003; Sekuler et al. 2000). These changes in gaze behavior may, however, not always accompany changes in motor performance (Chapman and Hollands 2006); Chapman and Hollands (2006) compared eye movements during gait in healthy older and younger adults and reported gaze differences even when comparable MT’s were achieved. This suggests that age related changes in cognitive processes do occur in the absence of physical decline. This potentially challenges the efficacy of using mental practices techniques such as AO and MI for motor relearning in older adults. We are aware of only two published studies (Heremans et al. 2012a, b) that have reported the eye movements of an older adult control group (>60 years) during the AE and MI of a wrist flexion/extension task. In both studies, the number of fixations and inter-fixation amplitude was found to be congruent between the two conditions. Based on these findings Heremans et al. suggested that MI ability was preserved in older adult populations. Whilst the congruent eye movements do suggest cognitive organization of the action was similar between AE and MI, the absence of a younger healthy control group makes it difficult to identify to what extent, if any, the performances were influenced by age-related changes.

Many consider age-related changes to manifest from 65 years onwards. Supporting this assumption, numerous studies have demonstrated performance breakdown when extremes of the adult age continuum are compared young adults (20-25 years) and older adults (70+ years). While overt age-related changes in motor tasks (for example an increase in reaction time) may not be apparent until over the age of 65, older adults have to invest additional cognitive effort to achieve comparable reaction times with young adults (Chapman and Hollands 2006; Seidler et al. 2010). Thus, the cognitive techniques used to compensate for age-related changes may mask the observable onset of age-related decline. Indeed in a recent review examining mental processes and aging, Saimpont et al. (2013) provided evidence of age-related changes in participants of 55 years and older. Others (Salthouse 1996) have also suggested that cognitive processes such as working memory and attentional control, arguably processes that are at the core of mental practice, begin to decline before the age of 50. These findings suggest it may be pertinent to investigate the influence of aging in a slightly younger population than traditionally recruited.

The primary aim of this study was to compare the AE, MI and AO of upper limb movement between healthy young and early aging adults. Specific eye movements provided the primary dependent variables and additional measures (MT and self-reports) were used to triangulate the data and confirm participant compliance in the covert tasks. Based on the findings of others (Flanagan and Johansson 2003; Heremans et al. 2008), and the concept of shared neural networks in motor simulation (Jeannerod 1994), we hypothesized that the gaze strategy executed in AE would be preserved in MI and AO. Due to age-related slowing, we expected the MT in AE to increase in the older group but, based on the conflicting findings to date, we made no predications regarding whether the MT in MI would increase or decrease compared to the physical MT. We hypothesized that MT, in MI and AE would be influenced by target size (Decety et al. 1989; McCormick et al. 2013).

## Methods

### Participants

A sample of 16 healthy participants was equally split into two age groups, old (mean age = 59 ± 7 years, 7 females) and young (mean age 30 ± 11 years, 7 females). Prior to testing it was confirmed that all participants: had normal or corrected to normal vision; were righted handed (old group, = 94.75 ± 4.35; young group, 95.80 ± 4.85 years (Edinburgh Handedness Inventory; Oldfield 1971); had at least average imagery ability (old group, visual imagery = 34.63 ± 6.37, kinesthetic imagery = 33.25 ± 10.01; young group, visual imagery = 31.88 ± 10.35, kinesthetic imagery = 34.25 ± 5.23 (Movement Imagery Questionnaire – Revised, MIQ-RS; Gregg et al. 2010). Two participants in the older group were retired but still physically active, reporting cumulative walking of at least 60 minutes each day. The remaining participants were office workers or similar. All participants volunteered to take part in the study, were naive to the hypotheses being tested, and supplied written informed consent prior to participation. Experimental procedures were approved by the local ethics committee of the host university.

### Apparatus

The tasks were performed using a calibrated (pre-experiment) tablet (ST2220T, Dell UK Inc.) and a hand held stylus (normal pen size and weight). The stylus movements were recorded at 50 Hz using DMDX (Forster and Forster 2003). The tablet had a spatial accuracy ± 2.5 mm, over 95 % of touchable area and a typical response time of 15 ms.

Eye movements were recorded with the Applied Science Laboratories Mobile Eye system (ASL; Bedford, Massachusetts) at a sampling rate of 30 Hz. The system has an accuracy of 0.5 ° of visual angle, a resolution of 0.10 ° of visual angle, and a visual range of 50 ° horizontal and 40 ° vertical. The Mobile Eye was recalibrated prior to condition (AE, MI, AO and control) using a 9-point grid presented on the tablet. A chin rest was used to restrict head movements. In this study a fixation was operationally defined as a stable gaze position (i.e. within 0.67 ° visual angle) that was maintained for at least 120 ms.

To promote intra-individual congruency between conditions, participants’ AE trials were covertly filmed using a Sony High Definition Handycam (HDR-HC7E). The camera was positioned directly above the participant and 186 cm from the floor. The personalized videos were then presented onto the tablet during each participant’s AO trials. The filming process was explained to participants during the final debrief session.

### Task

**Fig. 1:**
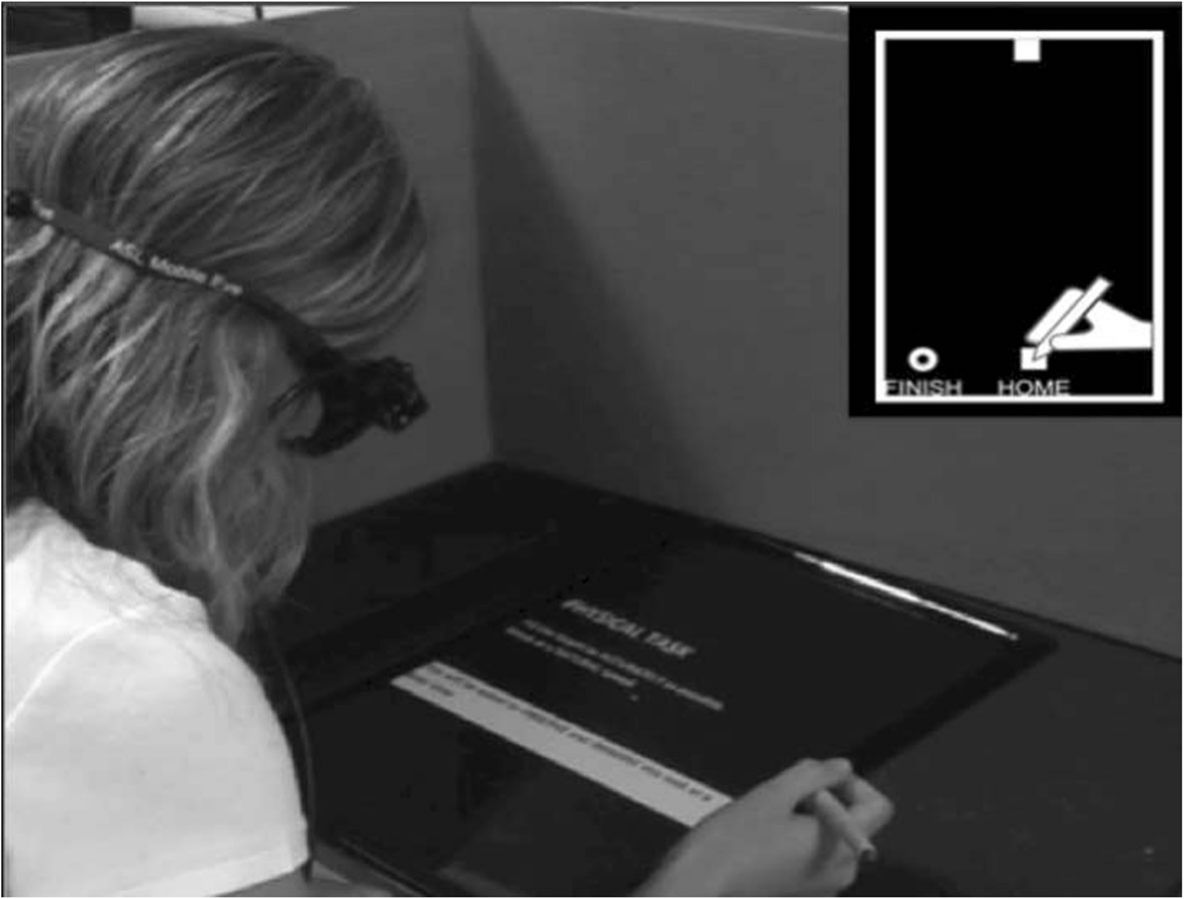
Top down schematic illustration of the experimental set up

Participants held the stylus in their dominant right hand and performed the Virtual Fitts’ Task (VFT, based on that used by McCormick et al. 2013) in three conditions: (i) AE; (ii) MI; and (iii) AO. In all conditions a HOME and FINISH button together with a TARGET square were presented on the tablet (see Fig. 1). The HOME and FINISH buttons were positioned approximately 200 mm away from the participant’s torso (midline). The TARGET was vertically aligned with the HOME button and the amplitude between the closest edges of the HOME and TARGET was constant (185 mm). Three TARGET squares of different sizes were used: small (4 mm^2^), medium (9 mm^2^) and large (20 mm^2^). According to the Fitts’ Law (Fitts 1954), the three target widths and the fixed inter-target distance lead to three indices of difficulty [ID = log2 (2A/W)]: respectively 6.5, 5.4 and 4.2.

In AE and MI, the HOME button was tapped to begin the task. In AE, participants physically moved the stylus to the TARGET, back to HOME and then to FINISH. In MI, the same action was imagined without any concomitant movement. The MT, the time from when the stylus left the HOME button until it pressed the FINISH button, was recorded in both AE and MI. In AO, the participants held the stylus and observed a recording of their own AE, presented onto the tablet as a video clip on the tablet.

To ensure a maximally homogeneous task across all participants a series of instructions were issued. In AE, participants were requested ‘to move the stylus as quickly as possible but not to risk improving speed at the expense of accuracy’. Participants were informed that ‘two or more false starts or target misses during any block would result in that block being restarted’. In MI, participants were instructed to ‘imagine the task from a first person egocentric, visual orientation’. To control the MI, a brief script was recited by the experimenter which described the scenario and the imager’s inner response to scenario (Lang 1979): ‘see yourself accurately reach the square target, as if you were actually performing the movement’ and ‘feel your grip on the stylus, feel the muscles in your upper arm contract, feel your arm extend as you perform the movement’. Participants were requested to refrain from any upper limb movement in this condition. In AO, the participants were instructed to remain stationary and to ‘observe the action with the intention to imitate it at a later time’.

### Experimental procedure

**Fig. 2:**
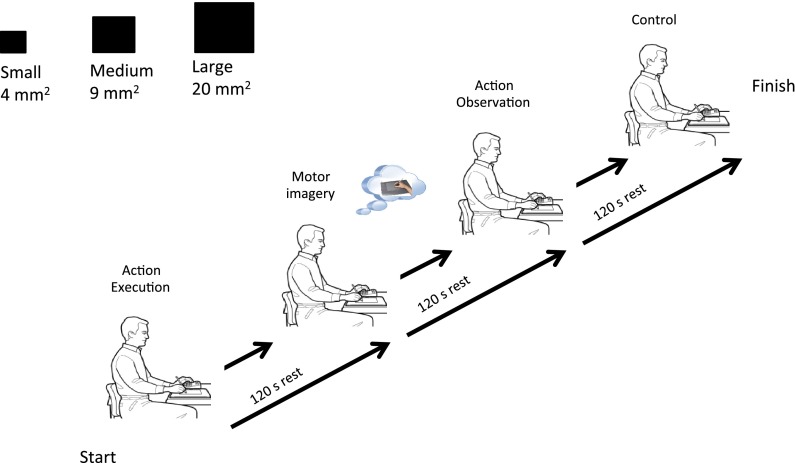
Example experimental series to the small target. Each series began with action execution, followed by imagery, observation and control (counterbalanced). The series were repeated (counterbalanced) for all three target sizes

Participants were fitted with the eye tracking system and initially performed a single habituation block of the VFT using a target that was a different size (15 mm^2^) to the experimental tasks. Participants were then assigned to one of three starting series defined by target size (small, medium, large). Each series began with one block (11 repeated reach tasks) of AE, followed by one block of each of the other conditions (i.e. MI, AO, and Control, counterbalanced; see Fig. 2). Preceding the covert conditions with AE was a necessity to maintain equivalent self-referent representations based on stored memories of a prescribed task (Borst and Kosslyn 2008). Each block consisted of eleven repetitions of the task followed by a 2-minute rest. At the end of the experiment each participant was debriefed fully and manipulation checks were performed to confirm participant compliance in the covert tasks. An in-house questionnaire, using a 7-point Likert-type scale (similar to the MIQ-RS), was used to rate the ease/difficulty associated with their visual and kinesthetic performance in MI and their active visual engagement and kinesthesis in AO*.*

### Control

To ensure that the eye movements in the simulation conditions did not reflect random oculomotor behavior a control condition was included. In this condition the TARGET, HOME and FINISH buttons were presented on the tablet and participants were instructed to count back slowly from 100. After 60 s (a time equivalent to the mean time spent performing a complete block of repeated tasks in AE) the participants were asked to rest.

### Gaze analysis

The eye movement data was analyzed using Gazetracker software (Lankford 2000). ‘Look-zones’, areas equivalent to the target plus a tolerance: small = 8 mm^2^; medium = 7 mm^2^; large = 6 mm^2^, were determined (McCormick et al. 2013). The tolerance, determined a posteriori, accommodated for drift, compressions, expansions and individual gaze behavior preference (Laeng and Teodorescu 2002; Liman and Zangemeister 2012). The spatial and temporal parameters of fixations located within the look-zones and the primary eye movement amplitude, calculated as the distance of the location of the first fixation from the HOME button following task onset, were compared between conditions (Laeng and Teodorescu 2002; Richardson and Spivey 2000). The primary eye movement amplitude was calculated (in mm) as the distance of the location of the first fixation from the HOME button following task onset. The first trial in each block was discarded since pilot testing revealed MT in this trial to be more variable. In total, the data reflected 1440 trials: 16 (participants) × 3 (conditions; AE, AO and MI) × 3 (target size; small, medium and large) × 10 (task repetitions per block). For the gaze metrics, the mean values per block were determined and used in the statistical analysis. The data in the control conditions represented performance at a block level and therefore 144 trials were analyzed: 16 (participants) × 3 (conditions; AE, AO and MI) × 3 (target sizes; small, medium and large).

### Statistical analyses

To confirm participant compliance during MI, the MT was compared using a 2 (condition: AE, MI) × 3 (target size: small, medium, large) × 2 (age: young, old) repeated measures (RM) ANOVA. The temporal correspondence between AE and MI was further examined by calculating the within subject correlation coefficient for the older and younger groups (Bland and Altman 1995). This approach was used as the repeated observations prevented the data from being treated as a simple sample. This analysis would reveal the extent to which an increase in MT in AE was associated with an increase in MT in MI.

The total number of fixations was analyzed using 4 (condition: AE, MI, AO, control) by 3 (target size) by 2 (age) RM ANOVA. The control condition was included in this analysis to compare fixations in task related and task unrelated conditions.

The differences in fixation duration were compared using a 3 (condition: AE, MI, AO) × 3 (target size) by 2 (age) RM ANOVA. As with MT, the temporal correspondence of this metric between AE and AO, and AE and MI was further examined by calculating the within subject correlation coefficient (Bland and Altman 1995). This additional analysis would demonstrate to what extent an increase in fixation duration in AE was associated with an increase in fixation duration in AO, and MI. To complete the analyses the primary inter-fixation distance was also compared using a 3 (condition: AE, MI, AO) × 2 (size: large, small) × 2 (age) RM AVOVA. This variable is particular susceptible to task strategy, and controlling task strategy in aiming tasks, irrespective of task instructions, can be problematic (Gesierich et al. 2008). Under conditions of high ID individuals may adopt a strategy that focuses on speed, but under conditions of low ID individuals tend to adopt a strategy that focuses on accuracy (Lazzari et al. 2009). As the size of the medium target was not vastly different to either the large or small target, the focus of the strategy could have been either speed or accuracy. Indeed, contemporary research (Van Halewyck et al. 2014) has reported no age related differences in primary saccade amplitude when tasks that only differ by an ID of 1 bit are used. The medium target was therefore excluded from the analysis to remove any confound related to task strategy.

The Shapiro-Wilks and Levene’s tests were used to identify normal distribution and equivalent variance. Sphericity was assumed if Mauchly’s test of sphericity was > 0.05. Effect Sizes were calculated using partial eta squared values (η_p_^2^) and the alpha level for significance was set at 0.05. Pairwise comparisons were Bonferroni corrected. All data are presented as means and, where appropriate, Greenhouse-Geisser corrected.

## Results

All participants complied with the task requirements. Task noncompliance accounted for 16 trials (2 %) being retaken for the young group and 20 trials (3 %) being retaken for the older group.

### Chronometry measures

Main effects were found for condition (F_1, 14_ = 4.649, *p* = 0.049, η_p_^2^ = 0.249), target size (F_2, 28_ = 4.272, *p* = 0.024, η_p_^2^ = 0.234) and age (F_1,14_ = 5.694, *p* = 0.032, η_p_^2^ = 0.289). There were no significant interactions. Pairwise comparisons revealed MT was slower in MI (2.976 ± 0.993 s) compared to AE (2.538 ± 0.798 s). For target size, MT was quicker for the large target (2.571 ± 0.812 s) compared to the small target (2.887 ± 0.895 s, *p* = 0.035). Older participants took significantly longer to perform the task (3.178 ± 0.925 s) compared to younger participants (2.335 ± 0.801 s).

Based on Cohen’s guidelines (Cohen 1988), the within subject correlation analysis for MT in AE and MI indicated a statistically significant medium correlation for the young group (r = 0.478, *p* < 0.002) and a small, but significant, correlation for the old group (0.258, *p* = 0.037).

### Total number of fixations

There was a main effect for condition (F_1.872, 26.211_ = 29.811, *p* < 0.001, η_p_^2^ = 0.680), but not size (*p* = 0.366) or age (*p =* 0.310). There was a significant condition by age interaction (F _1.872, 26.211_ = 4.342, *p* = 0.026, η_p_^2^ = 0.237) and a significant condition by size interaction (F _2.704, 37.862_ = 3.427, *p* = 0.030, η_p_^2^ = 0.197).

Significantly more fixations were made in AE compared to all other states. Regarding the condition by age interaction, pairwise comparisons revealed that older individuals made more fixations in AE (17 ± 4) compared to AO (13 ± 3, *p* = 0.006), MI (13 ± 4, *p* = 0.029) and control (4 ± 5, *p* = < 0.001). Older participants also made significantly more fixations in AE compared to the younger group (13 ± 3, p = 0.019). For younger participants there was no significant difference in the number of fixations between conditions: AE (13 ± 3), AO (11 ± 2) and MI (12 ± 2) but significantly fewer fixations were observed in control (6 ± 7, *p* = 0.045). The number of fixations made during AO and MI was not significantly different between groups.

**Fig. 3:**
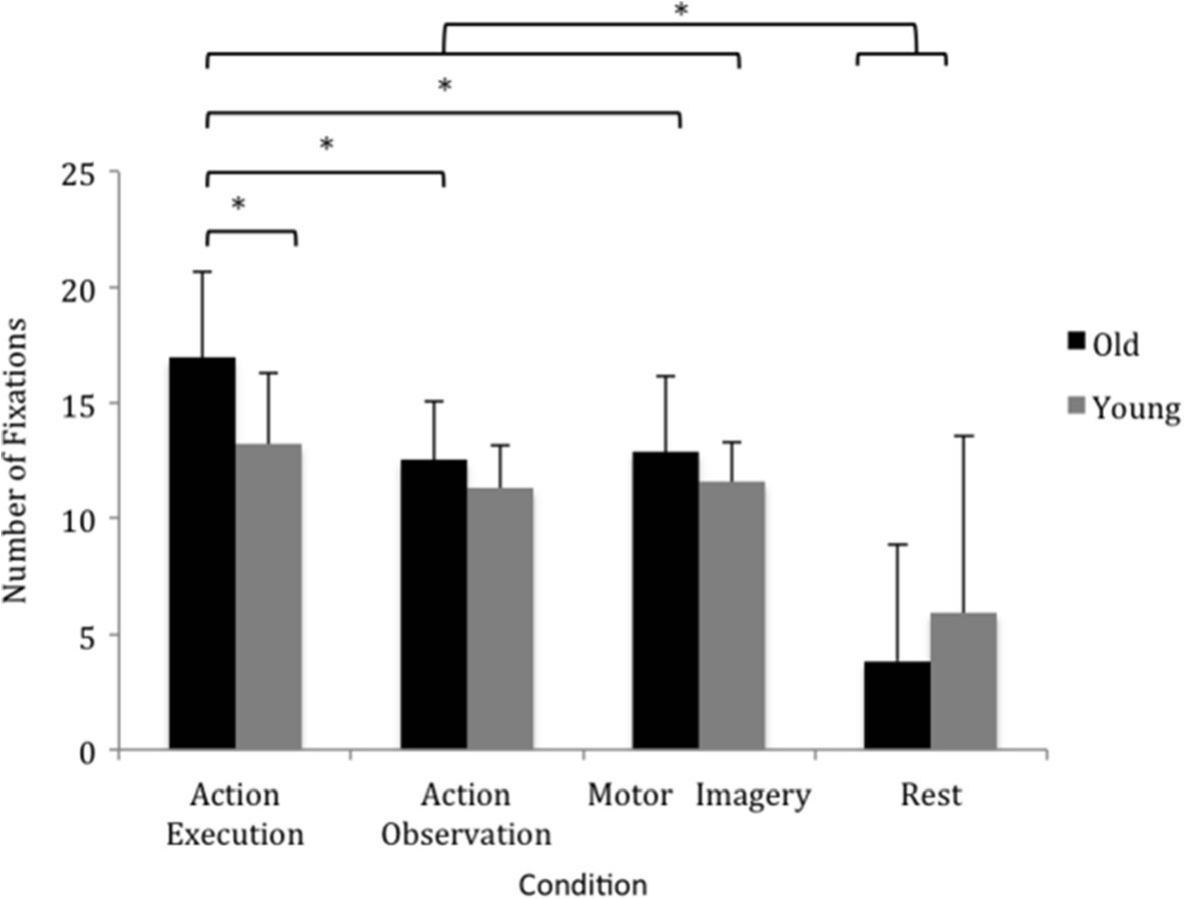
The number of fixations (mean ± SD) within the target look-zone. Each condition included 3 different target sizes and participants performed 10 reach actions to each target size. The data have been collapsed for target size

The condition by size interaction revealed that more fixations were made to the large target (6 ± 6) compared to the medium target (4 ± 7, *p* = 0.043) in the control condition only, see Fig. 3.

### Total fixation duration

**Fig. 4:**
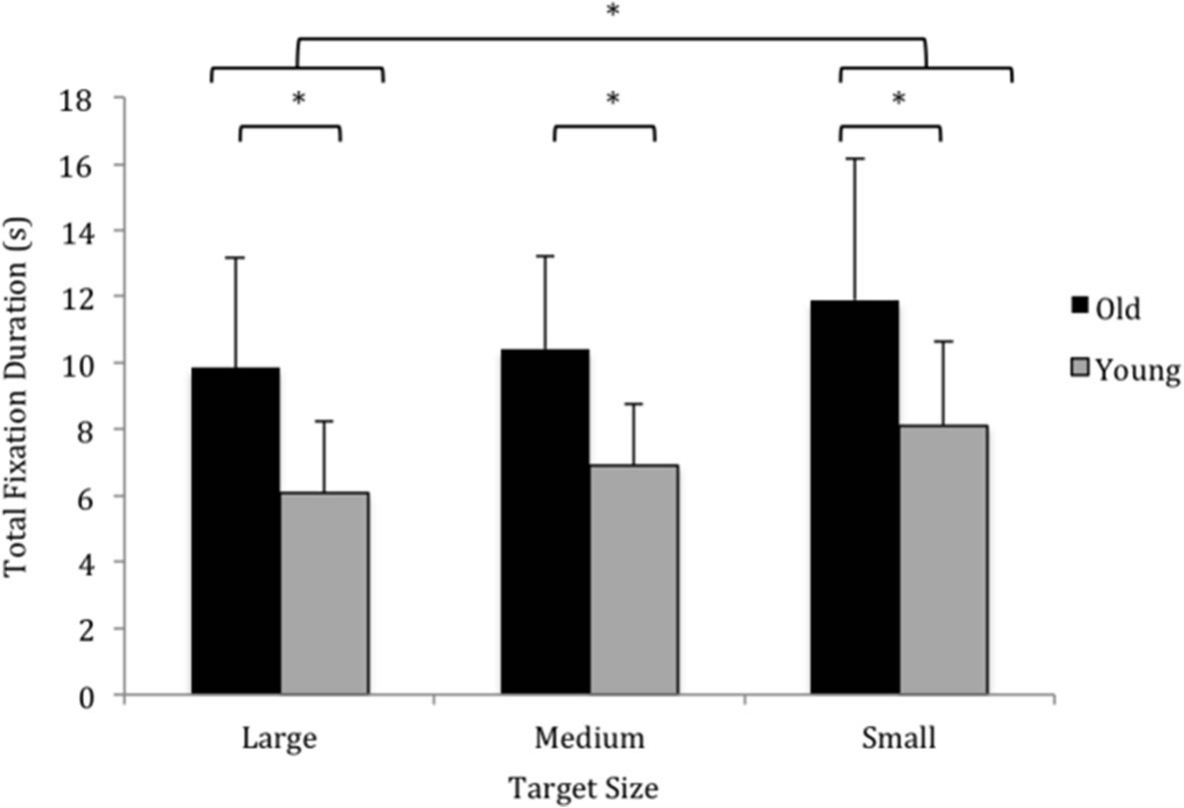
The total fixation duration during each block of 10 repeated reach actions. The data have been collapsed for condition as the ANOVA revealed no significant difference between AE, MI and AO

A main effect for size (F_1.339, 18.742_ = 9.734, *p* = 0.003, η_p_^2^ = 0.410) but not condition (F_1.356, 18.981_ = 1.239, *p* = 0.305) was found. Pairwise comparisons revealed that the total fixation duration was significantly less at the large target (7.968 ± 3.250 s) compared to the small target (10.010 ± 3.903 s), Figure 4. There was a main effect of age (F_1, 14_ = 7.351, *p* = 0.017, η_p_^2^ = 0.344) that indicated older participants, compared to younger participants, fixated the target look-zone for longer (10.721 ± 3.115 s vs 7.040 ± 1.910 s).

The within subject correlations for fixation duration in AE and AO, and AE and MI indicated a significantly medium-large correlation between AE and AO for the young (r = 0.447, *p* = 0.006) and older (r = 0.360, *p* = 0.01 l) group. The correlations between AE and MI were not significant (for either group).

### Primary eye movement amplitude

**Fig. 5:**
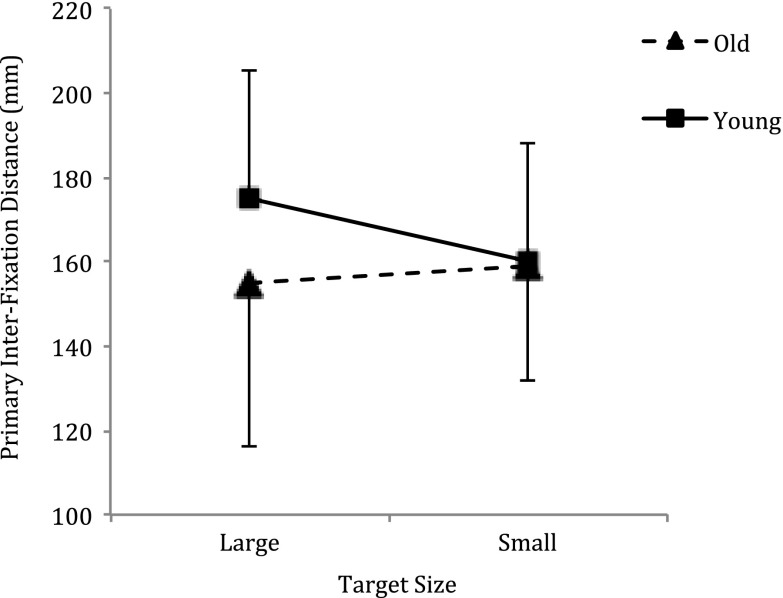
Primary eye movement amplitude (mean ± SD) per group for the large and small target sizes. The asterix indicates the significant difference in amplitude for the young group only

The RM ANOVA revealed a significant size by age interaction (F_1,14_ = 5.465, *p* = 0.035, η_p_^2^ = 0.281) (see Fig. 5). Simple effect analyses revealed the primary eye movement amplitude was greater to the large target (175.323 ± 29.918 mm) compared to the small target (159.914 ± 38.311 mm, *p* = 0.019) for the younger participants only. There were no significant main effects.

### Manipulation checks

Manipulation checks were completed post experiment to assess participants’ covert performance. In the older group, the visual component of MI was rated as “*somewhat easy to see*” (5.500 ± 1.195), and the kinesthetic component as “*somewhat hard to feel*” (3.500 ± 1.773). In AO, the visual component was rated as “*very easy to engage in*” (6.750 ± 0.707), and the kinesthetic component as “*very hard to feel*” (1.500 ± 2.828). In the younger group, the visual component of MI was rated as “*easy to see*” (5.625 ± 1.302), and the kinesthetic component as “*somewhat easy to feel*” (4.625 ± 1.408). In AO, the visual component was rated as “*very easy to engage in*” (7.000 ± 0.000, no variability in rated score), and the kinesthetic component as “*neutral (not easy or hard)*” (4.00 ± 2.928).

Participants preferred to perform AO compared to MI. 87.50 % (7 participants) of the older group, and 75 % (6 participants) of the younger group perferred this simulation condition.

## Discussion

Eye movements were measured in young and older adults to examine the influence of age on cognitive processes whilst participants physically executed, imagined and observed a goal directed action. The discussion is organized by dependent variable and self-reports have been included to supplement the findings. The chronometry data is discussed initially as this performance measure informs the interpretation of the data for the primary dependent variables.

### Chronometry measures

The chronometry results indicated that all participants complied with the task. As hypothesized, older individuals took significantly longer to physically execute the tasks, however in both age groups the mental and physical MT increased with target complexity. In both age groups the mental MT was longer than the physical MT and this may reflect the high degree of accuracy associated with the task (Guillot and Collet 2005). This group-wide increase in mental MT does not necessarily indicate impairment, but may reflect the different time constants of each condition; in MI the agent has to manipulate the image consciously: generate; inspect; maintain; and transform it (Dror and Kosslyn 1994) and this is predicted to introduce an additional time cost (Jeannerod 1997).

The correlation analysis revealed a weaker temporal relationship between AE and MI in the older group and this may indicate some loss of imagery accuracy. MT is considered to be derived, in part, from muscular force, proposed to be part of the coded motor representation (Jeannerod 1997). As the MI task involved no overt movement, there was no limb or object upon which to exert the planned force. Consequently, the level of force encoded in the motor command may have manifest as time in the covert states, with increases in felt force represented as increases in time (Decety et al. 1989). The inability of the older group to accurately represent MT in MI may therefore reflect a reduced ability to accurately predict muscular force as a result of a decline in sensorimotor control and a modified musculoskeletal system. In AE, this reduced ability may be counteracted by a greater reliance on proprioceptive and online visual feedback, which is partially absent in the MI condition (Klein et al. 2001; Smith et al. 1999; Poston et al. 2009; Chapman and Hollands 2006). The self-report data also appear to support these findings with older individuals rating the kinesthetic component of imagery as ‘*somewhat hard to feel*’ in comparison to the younger group who rated it as ‘*somewhat easy to feel’*. In addition, given that MI is primarily a top-down process, the weaker temporal relationship could also be explained by an age related decline in cognitive function (Seidler et al. 2010). In support of this suggestion some, but not all, imagery processes, such as the generation and maintenance of an image, are reported to become impaired with age (Dror and Kosslyn 1994).

### Number of fixations

Older participants made more fixations during AE compared to the younger group. The increase in the number of fixations suggests that the gaze strategy was less than optimal in this condition (Vickers 1996). The chronometry data would support this interpretation given that the older group also took longer when performing the task physically. Goggin and Meeuwsen (1992) suggest that older individuals place a greater emphasis on the posture phase of a pointing movement in order to maintain task accuracy. Since no difference was observed in gross endpoint error between the younger and older group, it is possible that the older participants invested more effort in this component of the task to maintain performance. This behavior would be supported by attentional control theory (Eysenck et al. 2007). Given that the hands and eyes are considered to be tightly coupled (Helsen et al. 1998), we suggest the additional fixations made by the older group represent the investment of this additional effort (Seidler et al. 2010).

In contrast to our hypothesis, the additional fixations executed during AE in the older group were not represented in the covert states. In MI and AO, fixations were fewer and similar in number to that of the younger group. These findings suggest that all participants adopted a similar gaze strategy in the covert conditions and both groups were equally proficient at the task. During physical movement, a crude feed-forward motor plan is generated and subsequently modulated by an error signal (the difference between the anticipated and actual position of the limb) determined through sensory feedback mechanisms (Desmurget and Grafton 2000). In the covert conditions the feedback is significantly limited and therefore the magnitude of the error signal maybe insufficient to modulate the motor plan. In these conditions the simulated action appears to be based only on the initial feed-forward motor plan; in all trials there was at least one fixation to the target location to assist in coding the coordinates of the movement trajectory. This explanation would support our earlier interpretation that the additional fixations observed during AE in the older group were related to the error correction phase concerned with stabilizing the hand at the target (Ghez et al. 2007). Indeed, direct evidence from studies of primate motor cortex suggest that the posture and movement phase of a reach action involve distinct processes (Kurtzer et al. 2005). A dissociation between the reach and grasp components of upper limb actions has been proposed (Jeannerod 1997; Grafton et al. 1996) and some authors (Edwards et al. 2003) report that in reach and grasp actions, the grasp component remains relatively robust to observational priming. The data from this study supports these claims by demonstrating the omission of specific gaze strategies during the sub components of the task in the covert conditions.

Some of our findings may appear to contrast with others. For example, Heremans et al. (2012a) reported no differences in the number and location of eye movements made by a healthy, older control group during overt and visually-cued MI. We propose differences in task demand may explain these conflicting findings. The Heremans et al. study required participants to physically and mentally perform a cyclic horizontal wrist flexion/extension action between two targets at two different indices of difficulty (4.5 and 5.3), at rate of 0.5 Hz. Arguably, this is a less demanding task compared to the present study, and, given that the participants typically made one eye movement per wrist movement during physical movement, we suggest that the initial feed forward motor plan was sufficient to guide the task. Given the earlier interpretation of our results, a similarity in the number of fixations during overt and covert movement would be expected.

For both age groups, the number of fixations within the look-zones was not influenced by target complexity, suggesting that task demand was compensated by other gaze behavior. Similar findings have been reported previously (McCormick et al. 2013; Heremans et al. 2012a).

Collectively, these data suggest that motor simulation in MI and AO may offer older individuals movement practice conditions that are not constrained by age-related decline. Of particular importance to practitioners is that covert states do not interpret the fine motor error corrections. The accuracy of the initial target fixation may, therefore, be critical to optimizing the mental practice benefits.

### Fixation duration

Fixation duration was influenced similarly across all conditions supporting the concept of shared neural substrate. Compared to younger individuals, older adults fixated for longer but displayed relatively similar increases in fixation duration with increases in target complexity. The longer fixation duration may be a result of age-related slowness associated with processing the visual information (Briggs et al. 1999) or a delayed arrival of the hand at the target due to functional loss (Kinoshita and Francis 1996; Smith et al. 1999).

The correlation analysis suggested that the congruency of fixation duration was enhanced between AE and AO in both groups. These data imply that the factors influencing the temporal allocation of an individual’s attention in AE influenced attention in AO similarly. The self-report data support this interpretation given that AO was rated as ‘*very easy to engage in’* by both the older and younger groups, whereas the visual dimension of MI was given a lower rating, either ‘*somewhat easy to see’* by the older group or ‘*easy to see’* by the younger group. In addition, 88 % of older adults and 75 % of younger adults reported a preference for AO in comparison to MI. Collectively, the findings suggest that older individuals perform better at, and prefer, AO. The temporal congruency between AE and AO, and the preference for using AO, maybe due to the common augmented feedback in these conditions. During AE and AO, the eye gaze strategy has been reported to work on a ‘just in time’ basis, where visual information is acquired and interpreted just at the point where it is required in the task (Flanagan and Johansson 2003; Hayhoe and Ballard 2005). This strategy is suggested to be employed to minimize the load of short term memory (Ballard et al. 1995). In contrast, in MI there is no augmented feedback and the image *is* the interpretation (Pylyshyn 2003). Thus the fixation duration (the time spent dwelling on a particular visual cue) is tightly governed by the evolution of the action in AO (as it is in AE), but is decoupled from the online action in MI. It is possible that under dynamic conditions individuals prefer using AO because it involves a more familiar and efficient eye motor strategy (Pylyshyn 2000).

It is possible that reduced congruency between AE and MI may have been due to an altered attentional focus. During the execution and observation of familiar well learned tasks individuals typically attend to the effect of an action rather than the limb movement required to achieve the action: they adopt a predictive, external attentional focus (Flanagan and Johansson 2003). Attending to the moving limb, although encouraged in acute movement rehabilitation, is reported to be detrimental to task performance outside of limits (McNevin et al. 2000; Hagemann et al. 2006). In accordance with Langian theory (Lang 1979), we facilitated MI in this study by including specific visual and kinesthetic statements, referred to as stimulus and response propositions. It is possible, therefore, that the kinesthetic response proposition “feel the muscles in your upper arm contract, feel your arm extend as you perform the movement” encouraged a more internal, specific attentional focus than the external, general focus adopted during overt movement. Indeed, other researchers (Calmels et al. 2006) have also reported that the presence of conscious kinesthetic sensations in imagery (sensations that are typically absent when the movement is physically performed) cause a temporal discrepancy between AE and imagined movement. These findings begin to highlight the multifarious influences on MI and the importance of the delivery instructions. Understanding how to control but not constrain imagery for effective therapeutic use should be explored in future research.

Taken together these findings demonstrate that although older adults fixate for longer, their visual information processing behavior is influenced in a manner similar to younger adults. There is some indirect evidence of neural sharedness across conditions (all conditions were influenced by target size), however the enhanced congruency between AO and AE suggests that dynamic, rather than static, visual cues activate additional shared processes in these conditions. In view of these findings, including dynamic visual cues in MI or performing MI simultaneously with AO, may support a more effective gaze strategy may enhance the efficacy of MI as a movement practice tool (Vogt et al. 2013). A similar approach of using augmented visual feedback to correct suboptimal gaze during AE has been demonstrated in the sports and clinical domains (Crowdy et al. 2002; Hagemann et al. 2006).

### Primary eye movement amplitude

The amplitude of the primary eye movement is considered to reflect the unmodified motor representation and is one of the first movements to be executed once a motor action has been programmed (Abrams et al. 1990). In this study, the primary eye movement amplitude was differentiated by target size in the young group only. Specifically, increases in primary eye movement amplitude accompanied decreases in task complexity in AE, MI and AO. This suggests that the younger group generated a motor representation based on task constraints. In contrast, the primary inter-fixation distance was not differenced by target size in the older group and this suggests that these individuals were either unable to modulate the amplitude, perhaps through functional loss, or executed a more conservative amplitude as a compensation mechanism.

In relation to the first suggestion, hypometric saccades (smaller inter-fixation distances) are reported to occur in senescence (Huaman and Sharpe 1993; Irving et al. 2006). In this study however, the mean amplitude of the primary inter-fixation distance executed by the younger adults was within the range reported to still be achievable by healthy older adults. Furthermore, Heremans et al. (2012a) reported that a healthy, older adult control group adapted their primary inter-fixation distance to different target distances in a horizontal aiming task. It therefore seems unlikely that the older adults in this study were unable to voluntary adjust the amplitude to reflect the target complexity. A more likely explanation is that amplitude was constrained in the large target task as a compensation mechanism. The hand movement amplitude of older adults is suggested to behave differently to that of younger adults. The relative distance travelled in the primary sub-movement is reported to be substantially less in older adults, with the movement highly influenced by accuracy constraints (Ketcham et al. 2002). Altered muscle activation patterns and deficits in force modulation have been cited as possible causes for these age-related changes (Darling et al. 1989). Given that hand movement amplitude is suggested to be closely coupled with eye movement amplitude (Cotti et al. 2007), the conservative primary eye movement amplitude may be a mechanism used to compensate for suboptimal hand movements by providing greater control during the terminal phase of the movement. Chapman and Hollands (2006) suggested that the central nervous system of older adults requires additional time to pre-plan movement and, to compensate for this, older individuals adopt a less than optimal gaze strategy the prioritizes the planning the movement.

The inter-fixation distance adopted by each group in AE was preserved in AO and MI. This pre-programmed part of the movement therefore appears embedded within the motor representation and, as such, may lend itself well to corrective covert practice. Cotti et al. (2007) demonstrated that the adaptation of voluntary saccades (inter-fixation distances) to targets generalizes to hand pointing movements, specifically the amplitude of the hand movement increases with the amplitude of the saccade. If this is true then executing larger saccades (i.e., increasing the inter-fixation distance) during the AO and MI of reach movements may offer opportunities to improve the physical execution of these tasks in older individuals.

### Control

For both groups, there were significantly fewer fixations to the target look-zone during the control condition. In addition, the number of fixations was differentiated by target size in the control condition but not in AE, MI and AO. These findings highlight the difference between random eye movements made in the control condition and task related eye movements made in AE, MI and AO.

### Conclusion

There is evidence of age-related changes to gaze during AE but, due to the incomplete neural overlap, some of these changes are associated with processes that are not represented in the MI and AO. In this regard, the lack of neural sharedness has a facilitative effect and permits the practice of movement under conditions that are not influenced by functional loss. Some age-related changes in gaze are preserved across conditions, e.g., eye movements linked to movement planning. As these suboptimal eye movements appear part of the shared neural representation, opportunity exists to alter their behavior during simulated movement. For example, in sport psychology and clinical rehabilitation, augmented visual feedback has been used to correct suboptimal gaze strategies. Given the neural similarities between AE, AO and MI, the effects of using visual cues to correct ineffective gaze in AO and MI would be predicted to be retained in AE. Re-learning movement under these conditions, in absence of physical execution, reduces the risk of injury in this population. Regardless of age, healthy adults appear to perform more accurately, and prefer, simulation conditions that are supported by detailed visual information. This may be because the sensory information is better matched to AE under these conditions (Holmes and Collins 2001).
